# Treatment of long COVID complicated by postural orthostatic tachycardia syndrome—Case series research

**DOI:** 10.1002/jgf2.670

**Published:** 2023-12-18

**Authors:** Tomoya Tsuchida, Yuki Ishibashi, Yoko Inoue, Kosuke Ishizuka, Kohta Katayama, Masanori Hirose, Yu Nakagama, Yasutoshi Kido, Yoshihiro Akashi, Takehito Otsubo, Takahide Matsuda, Yoshiyuki Ohira

**Affiliations:** ^1^ Department of General Internal Medicine St. Marianna University School of Medicine Kawasaki Kanagawa Japan; ^2^ Department of Cardiology St. Marianna University School of Medicine Kawasaki Kanagawa Japan; ^3^ Department of Virology & Parasitology, Graduate School of Medicine Osaka Metropolitan University Osaka Japan; ^4^ Research Center for Infectious Disease Sciences, Graduate School of Medicine Osaka Metropolitan University Osaka Japan; ^5^ Department of Gastroenterological and General Surgery St. Marianna University School of Medicine Kawasaki Kanagawa Japan

**Keywords:** long COVID, postural orthostatic tachycardia syndrome, β‐Blocker

## Abstract

**Background:**

Coronavirus disease 2019 (COVID‐19) sequelae, also known as long COVID, can present with various symptoms. Among these symptoms, autonomic dysregulation, particularly postural orthostatic tachycardia syndrome (POTS), should be evaluated. However, previous studies on the treatment of POTS complicated by COVID‐19 are lacking. Therefore, this study aimed to investigate the treatment course of long COVID complicated by POTS.

**Methods:**

The medical records of patients who complained of fatigue and met the criteria for POTS diagnosis were reviewed. We evaluated the treatment days, methods and changes in fatigue score, changes in heart rate on the Schellong test, and social situation at the first and last visits.

**Results:**

Thirty‐two patients with long COVID complicated by POTS were followed up (16 males; median age: 28 years). The follow‐up period was 159 days, and the interval between COVID‐19 onset and initial hospital attendance was 97 days. Some patients responded to β‐blocker therapy. Many patients had psychiatric symptoms that required psychiatric intervention and selective serotonin reuptake inhibitor prescription. Changes in heart rate, performance status, and employment/education status improved from the first to the last visit. These outcomes were believed to be because of the effects of various treatment interventions and spontaneous improvements.

**Conclusions:**

Our study suggests that the condition of 94% of patients with POTS complicated by long COVID will improve within 159 days. Therefore, POTS evaluation should be considered when patients with long COVID complain of fatigue, and attention should be paid to psychological symptoms and the social context.

## Graphical abstract

Long COVID can present with various symptoms. Among these symptoms, autonomic dysregulation, particularly postural orthostatic tachycardia syndrome should be evaluated. This study aimed to investigate the treatment course of long COVID complicated by POTS.

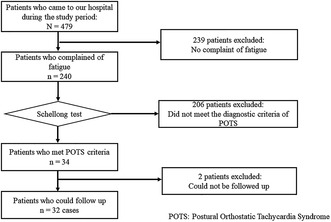

## 1 INTRODUCTION

Severe acute respiratory syndrome coronavirus 2 (SARS‐CoV‐2) has spread worldwide, with >760 million people infected.^1^ Recently, attention has been focused on coronavirus disease 2019 (COVID‐19) sequelae, also known as long COVID. The Centers for Disease Control and Prevention (CDC) defines long COVID as the onset of symptoms after 4 weeks of SARS‐CoV‐2 infection,^2^ whereas the World Health Organization defines the condition as follows: (i) usually 3 months from the onset of COVID‐19, (ii) with symptoms that last for ≥2 months, and (iii) cannot be explained by an alternative diagnosis. The common symptoms include fatigue, shortness of breath, and cognitive dysfunction, which generally impact daily functioning. New‐onset symptoms of long COVID may occur following initial recovery from acute COVID‐19, or symptoms may persist from the initial illness. In addition, the symptoms may fluctuate or relapse over time.^3^

The incidence of long COVID was reported to be 70% among hospitalized patients in China.^4^ However, with the emergence of the epidemic strains Delta and Omicron, the incidence was estimated to be 10.8% and 4.5%, respectively.^5^ Furthermore, symptoms were reported to persist after 1 year in 10%–30% of patients.^6^ Risk factors for long COVID include female gender, belonging to an ethnic minority, socioeconomic poverty, smoking, obesity, younger age, and having comorbidities.^7^ More than 50 symptoms of long COVID have been reported, with fatigue, headache, attention disorder, hair loss, and dyspnea as the most common manifestations. Other symptoms include postactivity polypnea, sweating abnormalities, nausea or vomiting, chest pains or discomfort, increased resting heart rate (HR), sleep disorders, flushing, and dizziness.^8^

Haloot et al.^9^ reported on the association between each symptom of long COVID and autonomic dysfunction. The mechanisms of autonomic dysfunction after COVID‐19 infection are considered multifactorial, and one of the main mechanisms is because of the direct effects of the virus. The idea is that persistent viremia causes autonomic dysfunction by inducing neuronal apoptosis and autonomic neuropathy. Postural orthostatic tachycardia syndrome (POTS) has been reported to be the most common autonomic dysfunction of long COVID; therefore, attention was drawn to POTS as a complication of long COVID.^10^, ^11^

POTS incidence ranges from 0.2% to 1% within the United States population, and this is based largely on clinical experience, suggesting that approximately 1–3 million individuals are affected in this population.^12^ Orthostatic symptoms include lightheadedness, visual blurring, tunnel vision, palpitations, tremulousness, and weakness (particularly in the legs). Other symptoms include fatigue, exercise intolerance, hyperventilation, shortness of breath, anxiety, chest pain, nausea, acral coldness or pain, concentration difficulties, and headache.^13^

Factors contributing to the development of POTS after COVID‐19 infection are hypovolemia, neurotropism, inflammation, and autoimmunity.^14^ Long COVID complicated by POTS was first reported by Miglis et al.^15^ and several reports have been published.^16^, ^17^, ^18^ Blitshteyn et al.^19^ reported a case series of 20 patients with long COVID complicated by autonomic neuropathy, 15 of whom had POTS. Additionally, the follow‐up of these patients for 6–8 months showed that only three patients could return fully to work.

Our facility started observing patients with long COVID in January 2021. As a university hospital, we treat patients through the Department of General Medicine and multidisciplinary cooperation, including specialists according to symptoms, nurses available to care for their mental health issues, and financial support from medical social workers. At the beginning of the outpatient assessment of patients with long COVID, we conducted Schellong tests on all patients, including those without fatigue, to evaluate autonomic dysfunction. However, patients who met the criteria for POTS were not those without complaints of fatigue. Kevi et al.^20^ reported in a survey of 779 patients with POTS in the United Kingdom that 91% of them complained of symptoms of fatigue. Therefore, we conducted the Schellong test only for those who complained of fatigue as a long COVID symptom.

If patients were diagnosed with POTS, lifestyle guidance, such as salt and fluid intake, was provided, and β‐blockers were prescribed as standard treatment.^21^, ^22^ In some cases, an otolaryngologist, psychiatrists, and social workers were consulted to treat autonomic dysfunction using epipharyngeal abrasion therapy (EAT), treat mental health issues, and provide financial support, respectively. As described above, reports of POTS diagnosed as a COVID‐19 complication exist. However, we could not find any report describing the detailed treatment course of an individual patient. Therefore, this study aimed to report the treatment course of long COVID complicated by POTS.

## 2 METHODS

### 2.1 Study design and setting

This descriptive study was conducted between January 2021 and May 2022 at the outpatient clinic of St. Marianna University Hospital (hereafter referred to as “our hospital”). The outpatient clinic for long COVID treats patients suspected of having long COVID who have been referred to our hospital from other clinics and hospitals.

### 2.2 Participants

Patients aged ≥15 years who visited our outpatient clinic for long COVID between January 18, 2021, and May 30, 2022, were included in the study. All patients fulfilled all of the following inclusion criteria: (1) confirmed SARS‐CoV‐2 infection through the antigen or polymerase chain reaction test, (2) the symptoms started until 3 months from COVID‐19 onset, (3) fatigue for ≥2 months after COVID‐19 onset, and (4) meeting the diagnostic criteria for POTS based on the Schellong test results. The diagnostic criteria for POTS were as follows: ① increase in HR by ≥30 beats/min within 10 min after standing upright in the absence of orthostatic hypotension (i.e., no sustained systolic blood pressure [BP] drop of ≥20 mmHg) and ② frequent symptoms of orthostatic intolerance during standing, with rapid improvement upon return to a supine position. The Schellong test was performed after 5 min of resting in a supine position. Furthermore, the BP and pulse rate were measured in the supine position; immediately after standing up; and after 1, 5, and 10 min.

### 2.3 Data collection

Patient data were obtained from medical records. These included gender, age, body mass index, number of days from COVID‐19 onset to initial hospital visit, number of medical institutions visited before visiting our hospital's outpatient clinic, the period from the initial hospital visit to the date of the last visit, underlying disease, and past medical history. Other data collected were main symptoms at the initial hospital visit, including physical (dyspnea, chest pain, and palpitations) and mental (lack of motivation, insomnia, anxiety, and low mood) symptoms; evaluation of employment or study status (i) continued with the job description or went to school same as that before the illness onset, (ii) continued with a job description different from that before the onset of illness or needed several days off per week from school (iii), leave of absence or school leave, and (iv) retirement; treatment details; whether symptoms improved after β‐blocker administration for 2 weeks to 1 month from the initial visit (second visit); the maximum change in HR (ΔHR) while resting in the supine and standing positions using the Schellong test on the initial, second, last visits; and the evaluation of fatigue at the initial and last visits. In addition, the performance status (PS) score was used to evaluate the degree of fatigue (Table 1),^23^ indicating the degree of chronic fatigue syndrome. We presented the fatigue score to the patients, and they selected it as a subjective symptom. In this study, we assessed the degree of fatigue score at the first visit, after initiating bisoprolol administration, and at the last visit within the study period. On the last visit, patients' continued use of bisoprolol was assessed to evaluate the need for bisoprolol treatment.

**TABLE 1:** Performance status score based on the degree of chronic fatigue syndrome.

PS	Criteria
0	You can live normally without fatigue You can act without restrictions
1	You can live and work normally but may sometimes feel tired
2	You can live and work normally but may sometimes need rest owing to general fatigue
3	You cannot work for several days per month owing to general fatigue
4	You cannot work for several days per week owing to general fatigue
5	You cannot live and work normally. Light work is possible, but rest at home is required for several days per week
6	You can do light work on days when your condition is good but need to rest at home 3–4 days per week
7	You cannot live normally or do light work but can take care of yourself
8	You can take care of yourself at the minimum level but often need help and lie down for more than 50% of the day
9	You cannot take care of yourself, always needing help and lying down all day long

Abbreviation: PS, Performance status.

### 2.4 Treatment options

As a standard treatment for POTS, patients were counseled to consume ≥10 g of salt and 2–3 L of water, except for those with hypertension and older people; instructed to wear elastic compression stockings up to the thighs; and prescribed bisoprolol, a β1‐selective blocker, once daily. We administered bisoprolol at a dose ranging from 0.625 to 2.5 mg and adjusted it based on symptoms and ΔHR. Treatment with Chinese herbal medicine, EAT performed in the otorhinolaryngology department (when POTS treatment is insufficient to improve symptoms), intervention for severe psychiatric symptoms, such as prescription of selective serotonin reuptake inhibitors (SSRIs) and consultation with a psychiatrist (if the patient has strong complaints of depression or anxiety symptoms), and intervention of social workers when financial support was necessary (when a patient's symptoms are severe to the extent of that they cannot work and has strong economic insecurity), were recommended in some cases. In addition, if patients complained of brain fog symptoms, such as cognitive dysfunction or difficulty understanding letters, we consulted the rehabilitation doctor to perform repetitive transcranial magnetic stimulation (rTMS).

### 2.5 Ethical considerations and approval

This study's protocol was approved by the Ethics Committee of Osaka Metropolitan University (Approval Number: 2020‐003).

### 2.6 Consent to participate

Adult (≥18 years old) and pediatric patients and their parents provided written informed consent for initial and follow‐up data collection as part of routine treatment. Informed consent was obtained from patients who visited our hospital before the study was approved by the ethics committee using an online opt‐out form.

## 3 RESULTS

**FIGURE 1:**
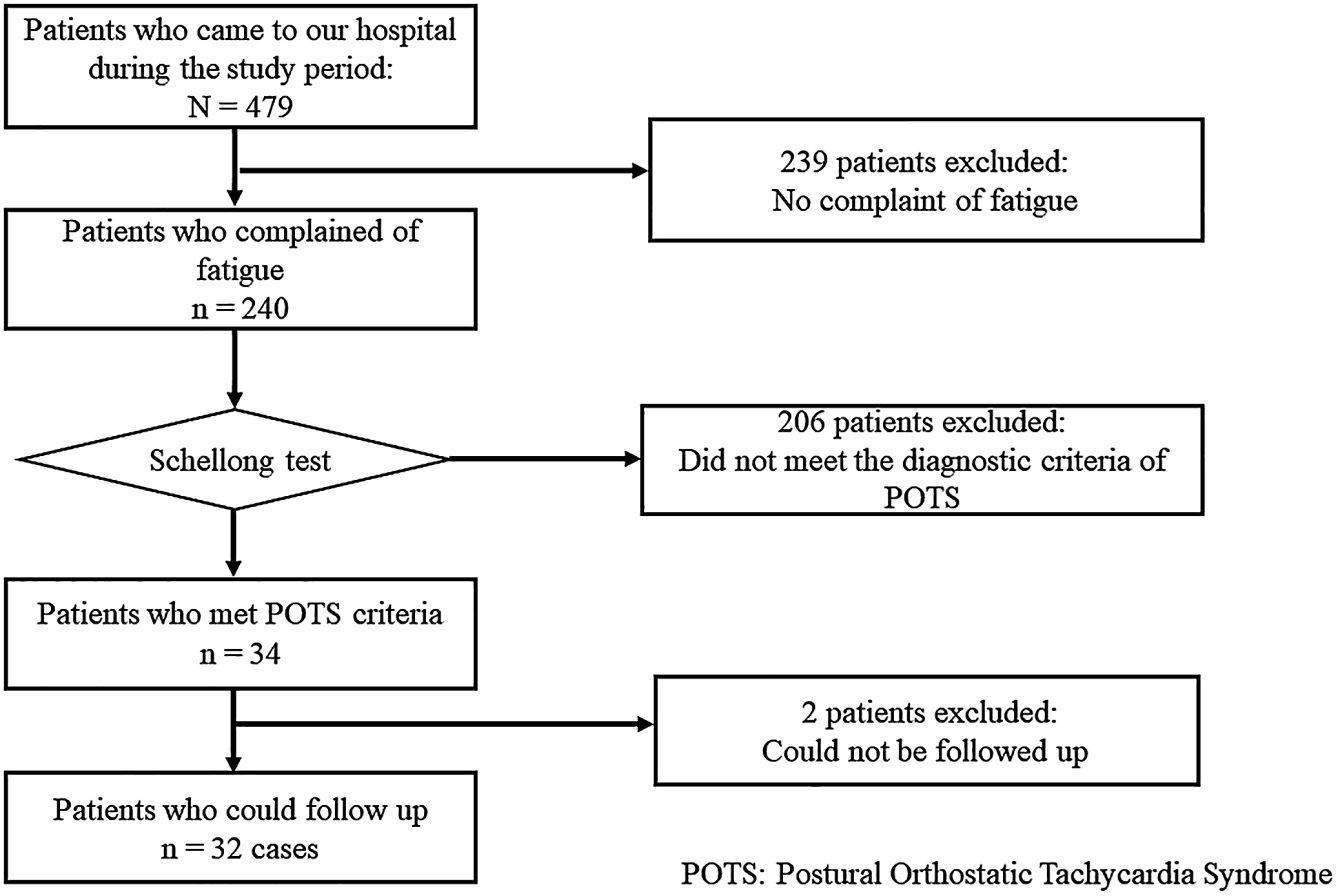
Flow diagram of the inclusion and exclusion criteria. Patients aged ≥15 years who visited our outpatient clinic for long COVID between January 18, 2021, and May 30, 2022, were included in the study. All patients fulfilled all of the following inclusion criteria: (1) confirmed SARS‐CoV‐2 infection through the antigen or polymerase chain reaction test, (2) the symptoms started until 3 months from COVID‐19 onset, (3) patients who remained fatigued for ≥2 months after SARS‐CoV‐2 infection, and (4) patients who met the diagnostic criteria for POTS based on the Schellong test results, and could be followed up for more than three times. COVID, coronavirus disease; POTS, postural orthostatic tachycardia syndrome; SARS‐CoV‐2, severe acute respiratory syndrome coronavirus 2.

Overall, 479 patients visited our outpatient clinic during the study period. Among them, 206 and 273 patients were males and females, respectively, with a median age of 43 (interquartile range [IQR]: 30–51) years. The median interval from COVID‐19 onset to the initial hospital visit was 120 (85–180.5) days. Of all patients, 240 (50.1%) complained of fatigue, and 34 (14.2%) met the criteria for POTS diagnosis. In addition, the treatment courses of 32 patients were evaluated after excluding 2 who had only two visits because of hospital transfer (Figure 1). These patients included 16 males and 16 females with a median age of 28 (IQR: 18.5–40) years. However, 206 patients complained of fatigue but did not meet the POTS criteria, and they included 90 males and 116 females with a median age of 44 (IQR: 32–50) years.

Table 2 shows the information and treatment courses of patients with POTS. The follow‐up period and the interval from COVID‐19 onset to the initial hospital visit were 159 (IQR: 90.5–264.5) and 97 (75–183) days, respectively. In addition, the maximum number of visits to medical facilities was four (*n* = 2 patients). Upon admission to the hospital, 29 patients complained of psychological symptoms, among the main symptoms other than fatigue. We refrained from recommending increased intake of water and salt to patients 5, 26, and 28 because of their high blood pressure or advanced age. After 2 weeks to 1 month of treatment (second visit), 19 patients were aware of the effects of bisoprolol treatment, while 13 were unaware. The median age of the 19 patients who were aware of the treatment effect was 31 (IQR: 25–42) years, and they had a median ΔHR of 43 (IQR: 35–45) at the initial visit. Furthermore, 6 of the 19 (31.6%) patients received a psychiatrist intervention or an SSRI prescription. In contrast, the median age of the 13 patients unaware of the treatment effects was 25 (IQR: 17–33) years, and they had a ΔHR of 36 (IQR: 32–40) at the initial visit. Seven of the 13 (53.8%) patients received a psychiatrist intervention or an SSRI prescription. Eight of the 12 (66.7%) patients who were on leave of absence or school leave required psychiatric intervention. Furthermore, 4 of the 19 (21.1%) patients who continued with their employment or went to school required psychiatric intervention. Other treatments included EAT and rTMS in 13 and 5 patients, respectively. Table 3 shows the ΔHR, PS score, and employment or study status at the initial and last visits.

**TABLE 2:** Patients' information and treatment courses.

Patient	Age (years)	Gender	BMI	Days from onset of COVID‐19 to initial visit	Medical institutions visited before visiting our hospital	Days from the initial hospital visit to the date of the last visit	Smoking	Underlying disease and past medical history	Physical symptoms other than fatigue	Psychiatric symptoms	Perceived effect after β‐blocker	Treatment medication other than β‐blocker	Other treatments	BP supine position	HR supine position	BP standing position	HR standing position	ΔHR at initial visit	ΔHR at second visit	Perceived effect after β‐blocker	ΔHR at last visit	Use of bisoprolol on final evaluation date	PS at initial visit	PS at last visit	Employment or study status at initial visit	Employment or study status at last visit
1	31	F	21	67	1	600	Never	Asthma	+	+	0	CHM, SSRIs	PT	119/83	88	131/83	136	48	0	0	13	×	6	0	LA	CS
2	25	M	18	69	1	58	Former smoker		+		0			113/58	63	98/66	113	50	26	0	10	×	4	0	CS	CS
3	40	F	18	76	1	301	Never			+++	×	CHM, SSRIs	EAT, PT	108/44	73	103/42	116	43	21	×	15	×	8	0	LA	CS
4	40	M	25	69	1	598	Former smoker		+	++	0	SSRIs	PT	125/76	63	137/107	107	44	25	0	36	0	8	3	LA	LA
5	60	M	22	93	2	196	Former smoker	Angina, Cerebral infarction, Hashimoto's disease, COPD	++	+++	0	CHM, Loflazepate		125/75	63	117/78	107	44	11	0	11	0	6	1	CS	CS
6	38	F	18	217	1	555	Never		+++	+++	0	Loflazepate	EAT, rTMS	106/55	68	108/82	100	32	13	0	4	0	2	1	CS	CS
7	32	M	24	74	1	76	Former smoker		+	++	0			124/80	54	160/100	90	36	5	0	−15	0	3	1	D	D
8	43	F	19	120	2	324	Never	Rheumatoid arthritis, Gastric cancer	++	++++	×	CHM, Midodrine	PT	109/72	101	101/69	136	35	12	×	14	×	6	1	LA	CS
9	18	M	17	132	2	294	Never		++	++++	0	Midodrine	PT	120/79	69	107/78	120	51	19	0	23	×	1	0	CS	CS
10	15	F	21	81	3	353	Never		+	+++	0	Lomerizine, Valproic acid	EAT	108/54	68	105/54	111	43	33	0	28	0	2	0	CS	CS
11	45	F	25	87	1	60	Former smoker		++		0	CHM		115/75	75	112/71	118	43	24	0	10	0	2	0	D	CS
12	45	M	23	78	1	59	Former smoker	Behcet's syndrome	+	+	0	CHM		120/80	64	115/84	105	41	9	0	36	×	1	0	LA	CS
13	31	M	26	99	1	88	Never	Cellulitis	+	++	0	Valproic acid, CHM	PT	125/79	74	126/78	109	35	23	0	15	0	3	3	CS	CS
14	17	M	20	307	4	61	Never				0	CHM	EAT	108/55	67	108/59	102	35	9	0	37	0	7	2	LA	CS
15	42	M	26	82	1	127	Former smoker			+++	0	CHM		118/62	63	118/63	98	35	18	0	15	×	3	1	D	CS
16	27	M	16	80	1	143	Never	Pneumothorax		+++	0			121/69	96	111/55	128	32	22	0	28	×	1	0	CS	CS
17	16	F	20	95	1	81	Never			+	×	Risperidone	PT	108/53	67	118/67	108	41	0	×	19	0	2	0	LA	LA
18	16	F	23	114	4	228	Never		+	+	×	CHM	EAT	108/58	56	123/65	89	33	23	×	48	×	1	0	D	CS
19	19	F	19	130	1	292	Never		+++	+++	0	PPI		103/44	61	112/50	106	45	23	0	10	×	4	2	D	CS
20	19	F	24	183	1	237	Never	Obsessive–compulsive disorder	+++	+++	×	Polycarbophil calcium	rTMS, PT	98/56	70	100/68	107	37	36	×	36	0	2	1	CS	CS
21	26	M	23	152	1	153	Never			++++	×	CHM		126/71	76	130/78	112	36	19	×	17	0	5	2	CS	CS
22	33	M	21	174	1	165	Never	Depression		++	×	SSRIs	EAT, rTMS, PT	131/72	59	129/68	91	32	31	×	24	×	6	1	LA	D
23	27	F	16	530	1	229	Never			+	×	CHM	EAT	103/53	58	114/71	100	42	24	×	30	0	2	1	D	CS
24	28	F	20	205	2	121	Never	Headache	+++	++++	0	Lomerizine, CHM		108/61	64	118/72	108	44	24	0	24	0	2	0	LA	CS
25	29	F	18	478	1	223	Never	Autonomic dysfunction, Infectious mononucleosis	+++	+	0		EAT, rTMS, PT	119/78	81	142/94	113	32	24	0	22	0	6	3	Retirement	Retirement
26	77	M	21	183	2	50	Never	Appendicitis, Enlarged prostate, Constipation, Hypertension	+		×	CHM		120/68	89	110/71	120	31	2	×	14	0	5	2	CS	CS
27	25	F	22	68	3	154	Never	Ulcerative colitis	++	+	×		EAT	126/76	107	122/67	143	36	9	×	13	0	6	3	LA	LA
28	28	F	36	64	1	154	Never	Hypertension		+++	0	CHM, Valproic acid	EAT, PT	145/108	67	155/94	106	39	28	0	31	0	5	0	D	D
29	46	F	21	434	1	164	Never	Bipolar disorder		+++	0	CHM	EAT, rTMS	111/74	68	106/72	114	46	20	0	5	0	4	1	D	CS
30	17	M	18	65	1	178	Never	Asthma	+	++	×	SSRIs	PT	99/63	67	95/64	107	40	29	×	23	0	5	2	LA	LA
31	17	M	19	289	1	98	Never			++	×	CHM	EAT	120/62	73	136/76	103	30	23	×	18	0	6	6	LA	LA
32	18	M	20	69	1	93	Never		++	+	×	SSRIs	EAT, PT	130/66	78	112/53	110	32	17	×	17	0	4	1	LA	D
Median (interquartile range)	28 (18.5–40)		21 (18.5–23)	97 (75–183)		159 (90.5–264.5)												38 (34–43.5)	21.5 (11.5–24)		17.5 (13–28)		4 (2–6)	1 (0–2)		

*Note*: ⚪: Effective/Use. ×: Not effective/No Use. D: Continued with a different job description or needed several days off per week to go school. CS: Continued with the same job description or went to school. The number of + represents the number of each of the physical and mental symptoms. Physical symptoms include dyspnea, chest pain, and palpitations, and mental symptoms include lack of motivation, insomnia, anxiety, and low mood.

Abbreviations: ADHD, Attention‐deficit hyperactivity disorder; CHM, Chinese herbal medicine; COPD, Chronic obstructive pulmonary disease; EAT, Epipharyngeal abrasive therapy; F, female; LA, Leave of absence or school leave; M, male; PT, Psychiatrist's treatment; rTMS, repetitive transcranial magnetic stimulation; SSRIs, Selective serotonin reuptake inhibitors.

**TABLE 3:** Summary of changes in maximum change in heart rate, performance status, and employment or study status between the initial and last clinic visits.

	Initial visit	Last visit
ΔHR median (IQR)	38 (34–43.5)	17.5 (13–28)
PS median (IQR)	4 (2–6)	1 (0–2)
Employment or study status		
Continued with the same job description or went to school	10 (31.3%)	22 (68.8%)
Continued with a job description different from that before the onset of illness or needed several days off per week from school	8 (25%)	4 (12.5%)
Leave of absence or school leave	13 (40.6%)	5 (15.6%)
Retirement	1 (3.1%)	1 (3.1%)

Abbreviations: PS, Performance status; ΔHR, Maximum change in heart rate.

The ΔHR (17.5 [IQR: 13–28]) and PS score (1 [IQR: 0–2]) improved on the last visit compared with those at the initial visit. Furthermore, the patients' employment or school status also improved, with (i) a decrease in the number of people who “took up a job different from that before the illness onset or needed several days off per week from school” and “leave of absence or school leave” and (ii) an increase in the number of people who “continued with the same job or went to school.”

## 4 DISCUSSION

This study evaluated the treatment course of long COVID complicated by POTS. In total, 32 patients were followed up for 159 days. Our analysis revealed that the number of patients who could work or go to school increased, and the PS score improved.

Two points are noteworthy in this study. First, at the initial visit, the 19 patients who were aware of the treatment's effect after the β‐blocker administration had a median ΔHR of 43 (IQR: 35–45), while the 13 who were unaware of the treatment's effect had a ΔHR of 36 (IQR: 32–40). This could have been because of the tachycardia‐suppressing effect of the β‐blockers, which might have resulted in the symptomatic improvement of previously unperceivable abnormal HR increases.

Second, 29 of the 32 patients had psychological symptoms. Previous reports have suggested that teenagers with POTS have only physical symptoms and no psychological symptoms^24^ and that SSRIs are ineffective in treating POTS symptoms.^25^ However, Shouman et al.^26^ suggested that patients with POTS after infection may have orthostatic symptoms exacerbated by disease‐related anxiety and poor physical health. Cameron et al.^27^ in their review of COVID‐19‐related POTS, found that a multidisciplinary approach, including the evaluation of both mental and physical health, is necessary.

In this study, many patients with POTS had psychiatric symptoms caused by financial and educational concerns because of their inability to go to work or attend school and anxiety about prolonged symptoms. In addition, patients' employment or school status at the last visit improved compared with that at the initial visit.

The natural history of POTS has been the subject of thorough investigation even before the COVID‐19 pandemic. A 1‐year follow‐up report of 58 patients (mean age, 27.9 years) with POTS diagnosis indicated improved overall symptoms and functional status (Autonomic Symptom Profile averaged over time), although only 20 patients did not meet the criteria for orthostatic HR in POTS at the end of the study.^28^ In a survey of 172 teenagers, only 33 (19%) respondents reported complete resolution of symptoms after 5.4 years; an additional 51% of respondents reported persistent but improved symptoms, and 28 (16%) experienced only intermittent symptoms.^24^ Table 3 shows that ΔHR improved from 38 (34–43.5) to 17.5 (13–28), and the PS score improved from 4 (2–6) to 1 (0–2) after a median follow‐up of 159 days from the initial visit. In this study, long COVID complicated by POTS symptoms may have improved more quickly than POTS unassociated with COVID‐19.

There are two possible reasons for this outcome. The first is the presence of autoantibodies. Various autoantibodies are reportedly produced after SARS‐CoV‐2 infection.^29^ Particularly, the production of adrenergic autoantibodies is believed to be an important factor in postinfection POTS.^30^ Originally, approximately 40% of POTS cases are reported to occur after some infectious diseases.^31^ However, all the patients in this study developed POTS following COVID‐19. Moreover, the production of autoantibodies following infection and the decrease in these autoantibodies because of the natural history of the infection may have influenced the early improvement of symptoms. Therefore, in the future, proving the presence of autoantibodies and evaluating whether they are associated with improvements in the clinical manifestations of POTS will be necessary.

The second reason is the effectiveness of various treatments. The treatment for POTS complications associated with long COVID at our hospital has included EAT since late 2021. EAT is a unique treatment in Japan and is believed to improve autonomic dysfunction caused by infections,^32^ which may have resulted in symptom improvement. In addition, when the patient had a history of depression or strong psychological symptoms, a psychiatrist was consulted early, and psychological treatment, including SSRI prescriptions, was provided. This may have contributed to early symptom improvement.

Seeley et al.^33^ conducted a detailed autonomic evaluation involving 33 patients with long COVID, 33 with POTS unassociated with COVID‐19, and 33 healthy individuals. Of the 33 patients with long COVID, 26 met the POTS diagnosis criteria. The degree of autonomic dysfunction was worse in patients with POTS unassociated with COVID‐19 than in those with long COVID. This finding aligns with that of our report, suggesting that the milder degree of autonomic dysfunction in patients with long COVID complicated by POTS could contribute to a potentially faster recovery. Although long COVID complicated by POTS may have a faster recovery than previous POTS, the risk of developing psychiatric symptoms because of a patient's social background should be carefully considered.

Several patients in this study had visited up to four medical facilities prior to our clinic and had not been diagnosed with POTS complications. Shaw et al.^31^ reported that POTS diagnosis took 2 years and involved seven doctors. In patients who complained of fatigue after COVID‐19, it was considered necessary to consider POTS complications.

### 4.1 Limitations

First, this was a single‐center descriptive study. Second, the outpatient clinic for patients with long COVID at our hospital provides only face‐to‐face care, and all patients were referred from neighboring medical institutions. Therefore, minor cases that did not require a referral and severe cases that could not be treated at our hospital because of severe symptoms and difficulty in performing physical activity might have been excluded from this study. Third, the Schellong tests were not conducted for patients with long COVID who did not complain of fatigue, and those with POTS who did not complain of fatigue were not included in this study. Finally, COVID‐19 variants differed by the time of infection. However, the differences could not be evaluated. Therefore, different results could have been obtained depending on the time of infection, and this is a subject for future investigation.

### 4.2 Conclusion

Our study reveals that an orthostatic test to evaluate POTS may be warranted for patients who complain of fatigue long after SARS‐CoV‐2 infection. Moreover, consideration for β‐blocker treatment may be particularly relevant for individuals exhibiting a substantial increase in HR during this test. Therefore, remaining attentive to the psychological symptoms that may manifest because of the unique social context experienced by patients dealing with POTS in the context of long COVID is crucial.

## FUNDING INFORMATION

This research received no grant from any funding agency in the public, commercial, or not‐for‐profit sectors.

## CONFLICT OF INTEREST STATEMENT

The authors have stated explicitly that there are no conflicts of interest in connection with this article.

## ETHICS STATEMENT

Ethics approval statement: This study was approved by the Ethics Committee of Osaka Metropolitan University Graduate School of Medicine (approval number: 2020‐003).

Patient consent statement: Adult (≥18 years old) and pediatric patients and their legal guardians provided written informed consent for collecting initial and follow‐up data as part of routine treatment. In addition, informed consent was obtained via an opt‐out form on the hospital's website for patients who visited our hospital before the ethics committee approved this study.

Clinical trial registration: None.

## References

[1] World Health Organization (WHO) coronavirus (COVID‐19) dashboard. 2022. https://covid19.who.int. Accessed 31 Jul 2023.

[2] CDC . Long COVID or post‐COVID conditions. 2022. https://www.cdc.gov/coronavirus/2019‐ncov/long‐term‐effects/index.html. Accessed 31 Jul 2023.

[3] World Health Organization . A clinical case definition of post COVID‐19 condition by a Delphi consensus. 2021. https://www.who.int/publications/i/item/WHO‐2019‐nCoV‐Post_COVID‐19_condition‐Clinical_case_definition‐2021.1. Accessed 31 Jul 2023.

[4] Huang C , Huang L , Wang Y , Li X , Ren L , Gu X , et al. 6‐month consequences of COVID‐19 in patients discharged from hospital: a cohort study. Lancet. 2021;397:220–232. 10.1016/S0140-6736(20)32656-8 33428867 PMC7833295

[5] Antonelli M , Pujol JC , Spector TD , Ourselin S , Steves CJ . Risk of long COVID associated with delta versus omicron variants of SARS‐CoV‐2. Lancet. 2022;399:2263–2264. 10.1016/S0140-6736(22)00941-2 35717982 PMC9212672

[6] Han Q , Zheng B , Daines L , Sheikh A . Long‐term sequelae of COVID‐19: a systematic review and meta‐analysis of one‐year follow‐up studies on post‐COVID symptoms. Pathogens. 2022;11:269. 10.3390/pathogens11020269 35215212 PMC8875269

[7] Subramanian A , Nirantharakumar K , Hughes S , Myles P , Williams T , Gokhale KM , et al. Symptoms and risk factors for long COVID in non‐hospitalized adults. Nat Med. 2022;28:1706–1714. 10.1038/s41591-022-01909-w 35879616 PMC9388369

[8] Lopez‐Leon S , Wegman‐Ostrosky T , Perelman C , Sepulveda R , Rebolledo PA , Cuapio A , et al. More than 50 long‐term effects of COVID‐19: a systematic review and meta‐analysis. Sci Rep. 2021;11:16144. 10.1038/s41598-021-95565-8 34373540 PMC8352980

[9] Haloot J , Bhavaraju‐Sanka R , Pillarisetti J , Verduzco‐Gutierrez M . Autonomic dysfunction related to oostacute SARS‐CoV‐2 syndrome. Phys Med Rehabil Clin N Am. 2023;34:563–572. 10.1016/j.pmr.2023.04.003 37419532 PMC10110930

[10] Reis Carneiro D , Rocha I , Habek M , Helbok R , Sellner J , Struhal W , et al. Clinical presentation and management strategies of cardiovascular autonomic dysfunction following a COVID‐19 infection – a systematic review. Eur J Neurol. 2023;30:1528–1539. 10.1111/ene.15714 36694382

[11] Raj SR , Arnold AC , Barboi A , Claydon VE , Limberg JK , Lucci VM , et al. Long‐COVID postural tachycardia syndrome: an American autonomic society statement. Clin Auton Res. 2021;31:365–368. 10.1007/s10286-021-00798-2 33740207 PMC7976723

[12] Vernino S , Bourne KM , Stiles LE , Grubb BP , Fedorowski A , Stewart JM , et al. Postural orthostatic tachycardia syndrome (POTS): state of the science and clinical care from a 2019 National Institutes of Health Expert Consensus Meeting – part 1. Auton Neurosci. 2021;235:102828. 10.1016/j.autneu.2021.102828 34144933 PMC8455420

[13] Freeman R , Wieling W , Axelrod FB , Benditt DG , Benarroch E , Biaggioni I , et al. Consensus statement on the definition of orthostatic hypotension, neurally mediated syncope and the postural tachycardia syndrome. Clin Auton Res. 2011;21:69–72. 10.1007/s10286-011-0119-5 21431947

[14] Chadda KR , Blakey EE , Huang CL , Jeevaratnam K . Long COVID‐19 and postural orthostatic tachycardia syndrome‐ is dysautonomia to be blamed? Front Cardiovasc Med. 2022;9:860198. 10.3389/fcvm.2022.860198 35355961 PMC8959615

[15] Miglis MG , Prieto T , Shaik R , Muppidi S , Sinn DI , Jaradeh S . A case report of postural tachycardia syndrome after COVID‐19. Clin Auton Res. 2020;30:449–451. 10.1007/s10286-020-00727-9 32880754 PMC7471493

[16] Ishibashi Y , Yoneyama K , Tsuchida T , Akashi YJ . Post‐COVID‐19 postural orthostatic tachycardia syndrome. Intern Med. 2021;60:2345. 10.2169/INTERNALMEDICINE.7626-21 34053992 PMC8355393

[17] Johansson M , Ståhlberg M , Runold M , Nygren‐Bonnier M , Nilsson J , Olshansky B , et al. Long‐haul post–COVID‐19 symptoms presenting as a variant of postural orthostatic tachycardia syndrome: the Swedish experience. JACC Case Rep. 2021;3:573–580. 10.1016/j.jaccas.2021.01.009 33723532 PMC7946344

[18] Gall NP , James S , Kavi L . Observational case series of postural tachycardia syndrome (PoTS) in post‐COVID‐19 patients. Br J Cardiol. 2022;29:3. 10.5837/bjc.2022.003 35747313 PMC9196071

[19] Blitshteyn S , Whitelaw S . Postural orthostatic tachycardia syndrome (POTS) and other autonomic disorders after COVID‐19 infection: a case series of 20 patients. Immunol Res. 2021;69:205–211. 10.1007/s12026-021-09185-5 33786700 PMC8009458

[20] Kavi L , Nuttall N , Low PA , Opie M , Nicholson L , Caldow E , et al. A profile of patients with postural tachycardia syndrome and their experience of healthcare in the UK. Br J Cardiol. 2016;23:33. 10.5837/bjc.2016

[21] Fu Q , Levine BD . Exercise and non‐pharmacological treatment of POTS. Auton Neurosci. 2018;215:20–27. 10.1016/j.autneu.2018.07.001 30001836 PMC6289756

[22] Miller AJ , Raj SR . Pharmacotherapy for postural tachycardia syndrome. Auton Neurosci. 2018;215:28–36. 10.1016/j.autneu.2018.04.008 29753556

[23] [Degree of fatigue and malaise according to PS (performance status) (PS is determined by the physician) In Japanese]. https://www.fuksi‐kagk‐u.ac.jp/guide/efforts/research/kuratsune/. Accessed 31 Jul 2023.

[24] Bhatia R , Kizilbash SJ , Ahrens SP , Killian JM , Kimmes SA , Knoebel EE , et al. Outcomes of adolescent‐onset postural orthostatic tachycardia syndrome. J Pediatr. 2016;173:149–153. 10.1016/j.jpeds.2016.02.035 26979650

[25] Mar PL , Raj V , Black BK , Biaggioni I , Shibao CA , Paranjape SY , et al. Acute hemodynamic effects of a selective serotonin reuptake inhibitor in postural tachycardia syndrome: randomized, crossover trial. J Psychopharmacol. 2014;28:155–161. 10.1177/0269881113512911 24227635 PMC3956655

[26] Shouman K , Vanichkachorn G , Cheshire WP , Suarez MD , Shelly S , Lamotte GJ , et al. Autonomic dysfunction following COVID‐19 infection: an early experience. Clin Auton Res. 2021;31:385–394. 10.1007/s10286-021-00803-8 33860871 PMC8050227

[27] Ormiston CK , Świątkiewicz I , Taub PR . Postural orthostatic tachycardia syndrome as a sequela of COVID‐19. Heart Rhythm. 2022;19:1880–1889. 10.1016/j.hrthm.2022.07.014 35853576 PMC9287587

[28] Kimpinski K , Figueroa JJ , Singer W , Sletten DM , Iodice V , Sandroni P , et al. A prospective, 1‐year follow‐up study of postural tachycardia syndrome. Mayo Clin Proc. 2012;87:746–752. 10.1016/j.mayocp.2012.02.020 22795533 PMC3538485

[29] Wang EY , Mao T , Klein J , Dai Y , Huck JD , Jaycox JR , et al. Diverse functional autoantibodies in patients with COVID‐19. Nature. 2021;595:283–288. 10.1038/s41586-021-03631-y 34010947 PMC13130511

[30] Kharraziha I , Axelsson J , Ricci F , Di Martino G , Persson M , Sutton R , et al. Serum activity against G protein‐coupled receptors and severity of orthostatic symptoms in postural orthostatic tachycardia syndrome. J Am Heart Assoc. 2020;9:e015989. 10.1161/JAHA.120.015989 32750291 PMC7792263

[31] Shaw BH , Stiles LE , Bourne K , Green EA , Shibao CA , Okamoto LE , et al. The face of postural tachycardia syndrome – insights from a large cross‐sectional online community‐based survey. J Intern Med. 2019;286:438–448. 10.1111/joim.12895 30861229 PMC6790699

[32] Imai K , Yamano T , Nishi S , Nishi R , Nishi T , Tanaka H , et al. Epipharyngeal abrasive therapy (EAT) has potential as a novel method for long COVID treatment. Viruses. 2022;14:907. 10.3390/v14050907 35632649 PMC9147901

[33] Seeley MC , Gallagher C , Ong E , Langdon A , Chieng J , Bailey D , et al. High incidence of autonomic dysfunction and postural orthostatic tachycardia syndrome in patients with long COVID: implications for management and health care planning. Am J Med. 2023;S0002‐9343(23)00402‐3. 10.1016/j.amjmed.2023.06.010 [In press]. PMC10307671 37391116

